# Current perspectives on cardiovascular outcome trials in diabetes

**DOI:** 10.1186/s12933-016-0456-8

**Published:** 2016-10-01

**Authors:** Oliver Schnell, Lars Rydén, Eberhard Standl, Antonio Ceriello, on behalf of the D&CVD EASD Study Group

**Affiliations:** 1 Forschergruppe Diabetes e.V., Munich, Ingolstaedter Landstrasse 1, 85764 Neuherberg (Munich), Germany; 2 Cardiology Unit, Department of Medicine K2, Karolinska Institutet, 171 76 Stockholm, Sweden; 3 Institut d’Investigacions Biomèdiques August Pi i Sunyer-IDIBAPS, Mallorca, 183, 08036 Barcelona, Spain; 4 IRCCS MultiMedica, Via Milanese, 300, 20099 Milan, Italy

**Keywords:** Cardiovascular risk, Diabetes, CVOT, Non-inferiority, Cardiovascular safety

## Abstract

Cardiovascular disease (CVD) is one of the most common diabetes-associated complications, as well as a leading cause for death in type 2 diabetes patients (T2D). Despite the well-known correlation between the two, up until the 2008 FDA industry guidance for licensing of new anti-hyperglycemic drugs, which required an investigation of cardiovascular outcomes (CVO) of glucose-lowering agents, only a few studies had looked into the relationship between glucose lowering drugs and cardiovascular (CV) risk. Thereafter, CVOT design has focused on non-inferiority short-term studies on high-risk patient populations aiming at capturing CV safety issues. Despite the wealth of information and useful data provided by CVOTs, this approach still suffers from certain limitations. The present review will condense the main results of the most recently completed CVOTs, reflect on the lessons learned, discuss on the issues presented by current CVOT design and offer some suggestions for improvement.

## Background

Among diabetes-related complications, cardiovascular disease (CVD) stands as the leading cause for mortality and adverse outcomes in patients with type 2 diabetes (T2D). More than 60 % die from CVD while an even greater proportion suffer serious CV-associated complications [1]. T2D implies a two to fourfold increase in the risk of coronary heart disease and a decreased life expectancy (6–7 years less) in comparison with people without diabetes [2]. Despite this clear correlation between diabetes and negative CV outcomes, it is still not clear whether glycemic control per se would have any effect on reducing CVD risk in T2D [3–6]. Moreover, CV safety of glucose-lowering drugs was not thoroughly investigated until the 2008 US Food and Drug Administration (FDA) [7] and subsequent European Medicines Agency (EMA) requirement [8] that all new therapies for diabetes undergo a rigorous assessment of CV safety through large-scale cardiovascular outcome trials (CVOT).

Before the publication of the FDA and EMA regulations, several trials assessing CV risks of glucose-lowering interventions had already been performed, if only with concerns in respect to design since they were aimed towards an improvement of glycemic control and outcome analysis [6]. For instance, in 1970 the first multicenter, head to head trial (University Group Diabetes Program) of T2D glucose-lowering treatments assessing CV outcomes was interrupted, as all oral drugs (tolbutamide, phenformin) seemed to increase CV risk in comparison to placebo or insulin [9–11]. However, this trial was grossly underpowered and therefore results often contested. Later, the 1977 UKPDS trial randomized patients to either standard or intensive diabetes care with either insulin, sulphonylurea or metformin. After 10 years, there was a significant reduction of MI risk and all-cause mortality in the intensive therapy group with any of the three drugs. However, the reduction of CV-associated risk was greater with metformin (39 % MI, 36 % all-cause) than with insulin or sulphonylurea (15 % MI, 13 % all-cause) [12]. A later meta-analysis of randomized trials using metformin found highly diverse results in terms of mortality risk increase/reduction as well as possible CV deleterious effects of a metformin/sulphonylurea combination [13], which were found to be greatly diminished 10 years after the end of the study and no longer statistically significant [14].

Other trials have found no differences in CV risk between glucose-lowering treatment interventions, as was the case for the HEART2D [15] trial, which compared basal and prandial insulin treatment strategies or the BARI 2D [16] trial, that compared insulin-sensitizing and insulin-providing treatment strategies in patients with T2D and CVD. However, the HEART2D trial was clearly underpowered, and a post hoc analysis seems to suggest a positive effect of controlling postprandial hyperglycemia in some subgroups of subjects, like older patients [17, 18]. The more recent ORIGIN trial, [19] which randomized patients with prediabetes and T2D patients with CVD risk factors to either insulin glargine or standard glucose control did also not find any differences to the primary CV outcome between treatment groups.

Several compounds have been suggested to increase CV risk in diabetes. For instance, several inter-related meta-analyses infer that rosiglitazone might raise MI and heart failure (HF) risk [20, 21]. Despite the RECORD trial [22, 23] only showed an excess HF risk without any conclusive results on MI, a meta-analysis including RECORD data still concludes that the high risk/benefit ratio of rosiglitazone does not support its use for diabetes treatment [21, 24]. The PROactive trial [25] on the CV safety of the addition to usual care of pioglitazone versus placebo found a slight trend toward a combined primary CV end-point—CVD and interventions in all vascular beds reduction—(10 % reduction, p = 0.095) and a significant 16 % reduction in the secondary end-point (MI, stroke, all-cause mortality). However, increased HF rates and a number of severe associated adverse events have hindered its use in daily practice [26, 27].

The requirements for CVOTs described in the aforementioned 2008 FDA guideline include, among others [28]:

- For outcome clinical trials, in order to exclude unacceptable CV risk, a two-sided 95 % CI upper boundary of 1.8 risk ratio (pre-approval) and/or 1.3 risk ratio (post-approval) for major adverse events (MACE) versus control group is required.
- To satisfy the new statistical requirements, CV event analysis might include a meta-analysis of all placebo-controlled, add-on (drug vs. placebo, plus standard therapy) and active-controlled trials, and/or an additional single, large, safety CVOT can be conducted. This, alone or in addition to other trials, needs to satisfy the upper bound mentioned above before approval.
- Patient selection should focus on high-risk populations, including those with advanced disease, elderly and those with renal impairment.
- Trials must include at least 2 years of CV safety data.
- A prospective independent adjudication of CV events in phase 2 and 3 studies must also be performed. These CV events include CV mortality, myocardial infarction (MI) and stroke, and possibly hospitalization for ACS, urgent revascularization and other end-points.

**Fig. 1:**
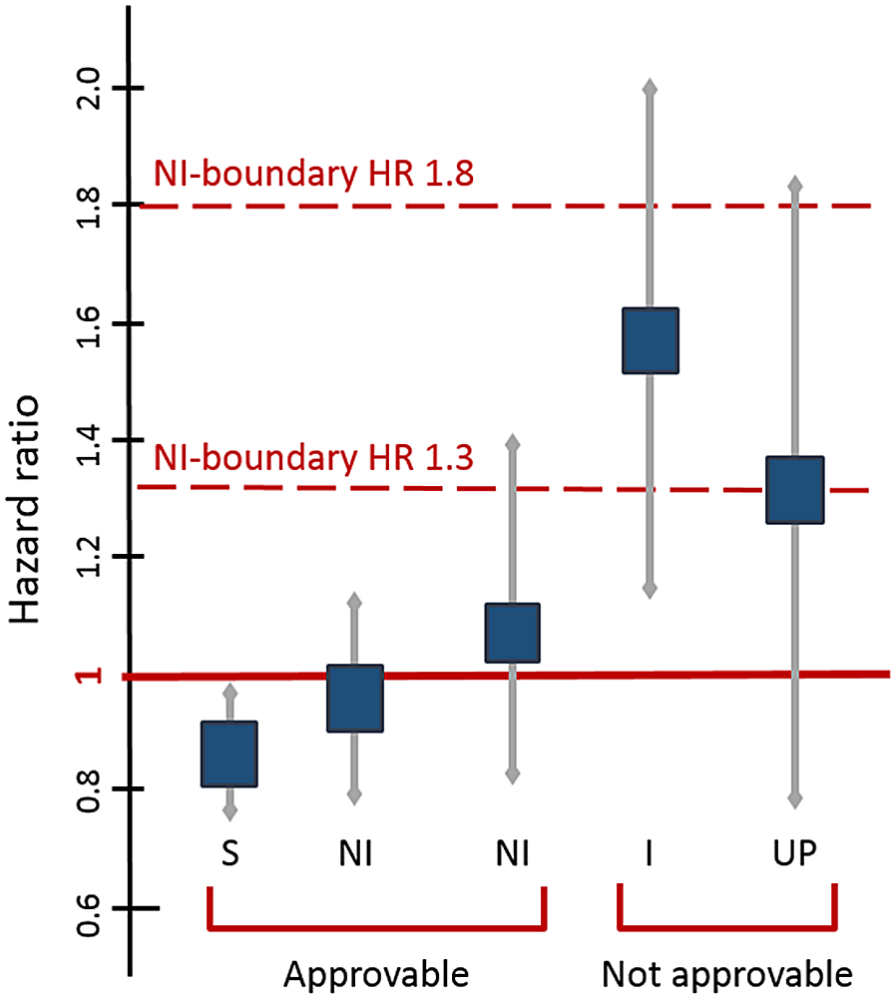
Confidence interval (CI) bars indicated by FDA guideline. Shown are five examples of hazard ratios (HR) and the upper limit of the 95 % CI of a development plan and regulatory consequences of each outcome. *S* superiority, *NI* non-inferiority, *I* inferiority, *UP* underpowered

Figure 1 includes a representation of possible scenarios for approval of new glucose lowering drugs depending on the hazard ratios (HR) for CV risk. An upper bound of the two-sided 95 percent confidence interval for the estimated increased risk above the non-inferiority boundary of 1.3 as well as underpowered studies prevents FDA approval. Surely, the need for full compliance with FDA/EMA requirements on CV safety for approval of new glucose lowering drugs has implied a significant increase of CVOTs in the last decade [28].

Results from early trials evaluating CV outcomes under glucose-lowering therapies could not ascertain a clear relationship between HbA1c target levels, hypoglycemia incidence and CV risk, despite a tendency for intense glucose control being beneficial in the long-term [6, 29–39]. Therefore, to avoid confounding results derived from glycemic values and the drugs themselves, CVOTs started after the 2008 FDA/EMA regulation have focused on maintaining glycemic equipoise, generally in the context of standard diabetes care [40].

In the present review, we will summarize the latest results of CVOTs on glucose-lowering agents started after the 2008 FDA Guideline as well as present an outline of ongoing CVOTs. Furthermore, we will review their influence on present glucose lowering therapy decision-making as well as comment on CVOT design limitations and potential venues for improvement.

### Summary of results of recently completed CVOTs

Since the FDA and EMA guidance request for CV safety for new antihyperglycemic drugs, over 15 medium/long-term CVOT have been initiated (see Table 1). From those, results for seven are already available while the remaining will be due by 2020 latest. In comparison to clinical trials on anti-hyperglycemic drugs performed prior to 2008, patient numbers have considerably increased (more than five times on average). So has the average number of countries per trial (1.6 times average), helping produce wider range data on other ethnic groups as well as in practice variation [6, 41, 42], while follow-up time remains on an average of 2.5 years [43–50].

**Table 1:** Basic characteristics of CVOTs started after 2008 FDA regulation

	Study status	Drug	Drug class	Intervention	Primary Outcome	N	Follow-up (years)	Start and estimated end date	Clinicaltrials.gov ID
SAVOR-TIMI53	Completed	Saxagliptin	DPP-4 inhibitor	Addition of saxagliptin vs. placebo to usual diabetes care	CV death, MI, or stroke	18,206	2.1	05.2010–05.2013	NCT01107886
EXAMINE	Completed	Alogliptin	DPP-4 inhibitor	Addition of alogliptin vs. placebo to usual diabetes care	CV death, MI, or stroke	5380	1.5	10.2009–06.2013	NCT00968708
TECOS	Completed	Sitagliptin	DPP-4 inhibitor	Sitagliptin vs. placebo	CV death, MI, UA, or stroke	14,724	3	12.2008–03.2015	NCT00790205
ELIXA	Completed	Lixisenatide	GLP-1 inhibitor	Lixisenatide vs. placebo	CV death, MI, UA, or stroke	6076	2.1	06.2010–02.2015	NCT01147250
EMPA-REG OUTCOME	Completed	Empagliflozin	SGLT-2 inhibitor	Empagliflozin 10 mg vs. empagliflozin 25 mg vs. placebo	CV death, MI, or stroke	7000	3.1	07.2010–04.2015	NCT01131676
LEADER	Completed	Liraglutide	GLP-1 inhibitor	Liraglutide vs. placebo	CV death, MI, or stroke	9340	3.8	08.2010–12.2015	NCT01179048
SUSTAIN-6	Completed	Semaglutide	GLP-1 inhibitor	Semaglutide 0.5 mg vs. semaglutide 1.0 mg vs. placebo	CV death, MI, or stroke	3299	1.99	02.2013–01.2016	NCT01720446
EXSCEL	Ongoing, not recruiting	Exenatide	GLP-1 inhibitor	Exenatide once weekly vs. placebo	CV death, MI, or stroke	14,000		06.2010–04.2018	NCT01144338
CAROLINA	Ongoing, not recruiting	Linagliptin	DPP-4 inhibitor	Liraglutide vs. placebo	CV death, MI, UA, or stroke	6000		10.2010–09.2018	NCT01243424
REWIND	Ongoing, not recruiting	Dulaglutide	GLP-1 inhibitor	Dulaglutide vs. placebo	CV death, MI, or stroke	9622		07.2011–01.2016	NCT01394952
ITCA650	Ongoing, not recruiting	Exenatide in DUROS	GLP-1 inhibitor	ITCA 650 (exenatide in DUROS) vs. placebo	CV death, MI, UA, or stroke	4000		03.2013–07.2018	NCT01455896
DECLARE-TIMI	Ongoing, not recruiting	Dapagliflozin	SGLT-2 inhibitor	Dapagliflozin 10 mg vs. placebo	CV death, MI, or stroke	17,276		01.2013–04.2019	NCT01730534
CARMELINA	Ongoing, not recruiting	Linagliptin	DPP-4 inhibitor	Linagliptin vs. placebo	CV death, MI, UA, or stroke	8000		07.2013–01.2018	NCT01897532
DEVOTE	Ongoing, not recruiting	Insulin degludec	Basal insulins	Insulin degludec vs. insulin glargine	CV death, MI, or stroke	7637		10.2013–09.2016	NCT01959529
MK-3102	Ongoing, not recruiting	MK-3102	DPP-4 inhibitor	MK-3102 vs. placebo	CV death, MI, UA, or stroke	4202		10.2012–12.2020	NCT01703208
Ertugliflozin trial	Ongoing, not recruiting	Ertugliflozin	SGLT-2 inhibitor	Ertugliflozin 5 mg vs. ertugliflozin 15 mg vs. placebo	CV death, MI, or stroke	3900		11.2013–06.2020	NCT01986881
TOSCA-IT	Ongoing, not recruiting	Pioglitazone	PPAR-γ agonists	Pioglitazone vs. sulfonylurea	Death, MI, stroke or coronary revascularisation	3371		09.2008–12.2018	NCT00700856
CANVAS	Ongoing, not recruiting	Canagliflozin	SGLT-2 inhibitor	Canagliflozin 100 mg vs. canagliflozin 300 mg vs. placebo	CV death, MI, UA, or stroke	4418		12.2009–06.2017	NCT01032629

Hereon we summarize the findings of all CVOTs started after the 2008 FDA guideline published to date, namely the SAVOR-TIMI, TECOS, ELIXA, EXAMINE, EMPA-REG OUTCOME, LEADER and SUSTAIN-6 trials [44–52]. Despite the focus on high-risk patients (a requirement for CVOT design), which poses a problem for extrapolation of results to the general patient population, the criteria for patient selection varied from trial to trial. For instance, age requirements of EXAMINE and EMPA-REG OUTCOME included all patients over 18 years old, while in other trials minimum age ranged between 30 and 50 years old. Cardiovascular risk also differed in each trial. While for most a preexisting CVD or CVD risk factors were necessary for enrolment, in the EXAMINE and ELIXA trials only patients already recovering from an acute coronary syndrome (ACS) were included in the study. For a detailed view on patient selection criteria, see Table 2. Moreover, an important aspect of CVOTs is that the evaluation of CV safety of the new glucose lowering drugs takes place in the background of diabetes and CVD standard care. This poses an important difference with respect to early trials like the UKPDS, performed before modern blood pressure reducing drugs; statins and an active attitude to coronary revascularization were part of routine care. Therefore, in Table 3 we have summarized the baseline concomitant medication of patients enrolled in trials started after 2008.

**Table 2:** Characteristics of patients enrolled in CVOTs referred to in the text

	Age	Diabetes type	HbA1c levels	Cardiovascular status	Prior antihyperglycemic treatment	BMI (kg/m^2^)
SAVOR-TIMI53	≥40	T2DM	≥6.5 %	CVD OR high CV risk	AHA	31.1
EXAMINE	≥18	T2DM	(6.5, 11.0 %)	ACS (15, 90) days before	AHA	28.7
TECOS	≥50	T2DM	(6.5, 11.0 %)	preexisting CVD	AHA	30.2
ELIXA	≥30	T2DM	≥7.0 %	ACS min. 180 days before	AHA	30.2
EMPA-REG OUTCOME	≥18	T2DM	(7.0, 10.0 %)	Preexisting CVD	Drug näive OR AHA	≤45
LEADER	≥50	T2DM	≥7.0 %	Preexisting CVD/cerebrovascular disease/vascular disease/renalORheart failure at ≥50 OR CV risk at ≥60	Drug näive OR AHA	32.5
SUSTAIN-6	≥50	T2DM	≥7.0 %	Preexisting CVD at ≥50 OR preCVD at ≥60	Drug näive OR AHA	31.1
EXSCEL	≥18	T2DM	(7.0, 10.0 %)		Specific AHA	
CAROLINA	≥40 ≤85	T2DM	(6.5, 7.5–8.5 %)	CVD OR specified diabetes end-organ damage OR age ≥70 years OR ≥2 specified CV risk factors		≤45
REWIND	≥50	T2DM	≤9.5 %	Preexisting vascular disease OR ≥CV risk factors	AHA	
ITCA650	≥40	T2DM	≥6.5 %	Preexisting coronary, cerebrovascular or peripheral artery disease		
DECLARE-TIMI	≥40	T2DM		High risk CV events		
CARMELINA	≥18	T2DM	(6.5, 10.0 %)	High risk CV events	Drug näive OR specific AHA	≤45
DEVOTE	≥50	T2DM	≤7.0 %	CVD OR renal disease OR ≥60 CV risk	Specific AHA	
MK-3102	≥40	T2DM	(6.5, 10.0 %)	Preexisting vascular disease		
Ertugliflozin trial	≥40	T2DM	(7.0, 10.5 %)	Preexisting vascular disease	Drug näive OR AHA	≥18
TOSCA-IT	≥50 ≤75	T2DM	(7.0, 9.0 %)		Specific AHA	20–45
CANVAS	≥40	T2DM	(7.0, 10.5 %)	Preexisting CVD OR high CV risk	Drug näive OR AHA	

*AHA* anti-hyperglycemic agents

**Table 3:** Concomitant medication at baseline in CVOTs referred to in the text

Concomitant medication @baseline	Antihyperglycemic medication N (%)			CV treatment N (%)					
	Insulin	Metformin	Sulphonylurea	Aspirin	Statins	Antiplatelet/anticoagulant	Beta-blocker	ACEI/ARB	Other anti-hypertensives
SAVOR-TIMI53	6757 (40.9)	11,094 (67.4)	6332 (38.5)	12,390 (75.2)	12,892 (78.3)	13,386 (81.3)	10,117 (61.4)	12,935 (78.5)	6730 (40.9)
EXAMINE	1605 (29.8)	3562 (66.2)	2503 (69.9)	4881 (90.7)	4866 (90.4)	5232 (97.2)	4411 (81.9)	4411 (81.9)	1197 (22.2)
TECOS	3408 (23.2)	11,966 (81.6)	6645 (45.3)	11,518 (78.5)	11,719 (79.9)	3167 (21.7)	9322 (63.5)	11,555 (78.8)	4961 (33.8)
ELIXA	2292 (37.8)	3834 (63.2)	1863 (30.7)	5726 (94.4)	5621 (92.6)	480 (7.9)	5119 (84.4)	5151 (84.9)	1327 (21.9)
EMPA-REG OUTCOME	2394 (34)^a^	3933 (55.9)^a^	1383 (19.6)	5990 (85)	5387 (77)	–	4537 (64)	5651 (80)	2114 (30)
LEADER	3905 (41.8)^a^	7136 (76.4)	4721 (50)	6523 (69.8)	6729 (72)	6322 (67.7)	5173 (55.4)	4761 (51)	920 (9.85)
SUSTAIN-6	1913 (58.0)	2414 (73.2)	1410 (42.8)	2108 (63.9)	2399 (72.8)	406 (12.3)	1894 (57.4)	1642 (49.8)	258 (7.8)
CAROLINA	–	4982 (82.5)	1728 (28.6)	3026 (50.1)	3872 (64.1)	–	2344 (38.8)	2664 (44.1)	1770 (29.3)
CANVAS	2171 (50.1)	3158 (72.9)	2032 (46.9)		3119 (72.0)	3073 (71.0)			

^a^Both mono and dual therapy

For clarity, results for each outcome will be split into distinct sections, starting by the primary composite end-point and then proceeding to each of the possible CV outcomes evaluated by these trials: MI, unstable angina (UA), CV death and HF. Finally, we will review a few other relevant safety end-points, namely: pancreatitis, hypoglycemia occurrence, and renal events/microvascular effects

- *Primary MACE composite end*-*point* Diverse individual elements are included in the primary composite end-point for each CVOT, as shown in Table 1. However, CV death, myocardial infarction and stroke are all common elements to primary composite CVOT end-points. In addition, the TECOS and ELIXA trials included hospitalization for UA in the primary MACE. Corresponding data in Table 4 shows that for saxagliptin (SAVOR-TIMI), sitagliptin (TECOS), lixisenatide (ELIXA) and alogliptin (EXAMINE) treatment, occurrence of the primary composite end-point did not differ from placebo groups, thus confirming non-inferiority of the new treatments in CV safety under the particular conditions of each of the trials. In the EMPA-REG OUTCOME trial, however, the primary outcome occurred in 10.5 % in the pooled empagliflozin group and in 12.1 % of the placebo group (empagliflozin group (HR 0.86; 95 % CI 0.74–0.99; p = 0.04 for superiority), demonstrating therefore not only non-inferiority versus placebo but superiority [49]. A similar result was observed in LEADER, where the primary outcome occurred in significantly fewer patients in the liraglutide group than in the control group (13 vs. 14.9 %; HR 0.87; 95 % CI 0.78–0.97; p = 0.01 for superiority), but only for patients with established CVD (subgroup analysis) [51]. It is important to note, however, that both in LEADER and EMPA-REG OUTCOME, the lesser occurrence in the primary composite end-point was largely driven by a reduction in cardiovascular mortality. Results from the recently published SUSTAIN-6 trial have also shown superiority for semaglutide versus placebo in the primary composite outcome (6.6 vs. 8.9 % of patients, respectively; HR: 0.74, 95 % CI 0.58–0.95; p < 0.001), however, in contrast to EMPA-REG OUTCOME and LEADER, results were not driven by a decrease of risk of cardiovascular death, but of non-fatal stroke occurrence (in 1.6 and 2.7 %, respectively (HR 0.61; 95 % CI 0.38–0.99; p = 0.04) [52].

**Table 4:** Comparison of outcome results from terminated CVOTs in comparison to placebo

Cardiovascular endpoints	SAVOR-TIMI53 [43, 45]		EXAMINE [47, 48]		TECOS [50]		ELIXA [44]		EMPA-REG OUTCOME [48, 49]		LEADER [51]		SUSTAIN-6 [52]	
	Class	Hazard ratio (95 % CI) p value	Class	Hazard ratio (95 % CI) p value	Class	Hazard ratio (95 % CI) p value	Class	Hazard ratio (95 % CI) p value	Class	Hazard ratio (95 % CI) p value	Class	Hazard ratio (95 % CI) p value	Class	Hazard ratio (95 % CI) p value
Primary composite MACE	CV death, MI, or stroke	1.00 (0.89-1.12) 0.99	CV death, MI, or stroke	0.96 (≤1.16) 0.315	CV death, MI, UA, or stroke	0.98 (0.89–1.08) 0.65	CV death, MI, UA, or stroke	1.02 (0.89–1.17) 0.81	CV death, MI, or stroke	0.86 (0.74–0.99) 0.04^a^	CV death, MI, or stroke	0.87 (0.78–0.97) 0.01	CV death, MI, or stroke	0.74 (0.58–0.95) <0.001/0.02^a^
Cardiovascular death	Primary end-point	1.03 (0.87–1.22) 0.72	Primary end-point	0.85 (0.66–1.10) 0.212	Primary end-point	1.03 (0.89–1.19) 0.71	Primary end-point	0.98 (0.78–1.22) 0.85	Primary end-point	0.62 (0.49–0.77) < 0.001	Primary end-point	0.78 (0.66–0.93) 0.007	Primary end-point	0.98 (0.65–1.48) 0.92
Myocardial infarction	Primary end-point	0.95 (0.80–1.12) 0.52	Primary end-point	1.08 (0.88–1.33) 0.47	Primary end-point	0.95 (0.81–1.11) 0.49	Primary end-point	1.03 (0.87–1.22) 0.71	Primary end-point	0.87 (0.70–1.09) 0.23	Primary end-point	0.86 (0.73–1.00) 0.046	Primary end-point	0.74 (0.51–1.08) 0.12
Stroke	Primary end-point	1.11 (0.88–1.39) 0.38	Primary end-point	0.91 (0.55–1.50) 0.71	Primary end-point	0.97 (0.79–1.19) 0.76	Primary end-point	1.12 (0.79–1.58) 0.54	Primary end-point	1.18 (0.89–1.56) 0.26	Primary end-point	0.86 (0.71–1.06) 0.16	Primary end-point	0.61 (0.38–0.99) 0.04
Hospitalization for unstable angina	Secondary end-point	1.19 (0.89–1.60) 0.24	Secondary end-point	0.90 (0.60–1.37) 0.632	Primary end-point	0.90 (0.70–1.16) 0.42	Primary end-point	1.11 (0.47–2.62) 0.81	Secondary end-point	0.99 (0.74–1.34) 0.97	Extended Primary end-point	0.98 (0.76–1.26) 0.87	Extended primary end-point	0.82 (0.47–1.44) 0.49
Hospitalization for heart failure	Secondary end-point	1.27 (1.07–1.51) 0.007	Extended Primary end-point	1.19 (0.90-1.58) 0.220	Secondary end-point	1.00 (0.83–1.20) 0.98	Secondary end-point	0.96 (0.75-1.23) 0.75	Secondary end-point	0.65 (0.50–0.85) 0.002	Extended Primary end-point	0.87 (0.73-1.05) 0.14	Extended primary end-point	1.11 (0.77–1.61) 0.57

	Event rate (%) active group	Event rate (%) active group	Event rate (%) active group	Event rate (%) active group	Event rate (%) active group	Event rate (%) active group	Event rate (%) active group							
	No. (%) p value	No. (%) p value	No. (%) p value	No. (%) p value	No. (%) p value	No. (%) p value	No. (%) p value							
Primary composite MACE	7.3	11.3	9.6	13.4	10.5	13.0	6.6							
Non-cardiovascular endpoints														
Renal event	2.0 % 0.46	0.9 % 0.88	1.5 %	1.6 %	5.2 %	5.7 %	3.8 %							
Acute pancreatitis	0.3 % 0.17	0.4 % 0.5	0.3 % 0.12	0.2 %	0.3 %^b^	0.4 % 0.44	0.54 %							
Hypoglycemia events	0.5 % 0.33	0.7 % 0.86	1.9 % 0.33	0.6 %	1.3 %	3.3 % 0.02	22.4 %^c^							

^a^Superiority test

^b^Average across all age ranges

^c^Severe hypoglycemia as defined by ADA
- *Cardiovascular death* In all terminated trials, treatment with the new agent did not increase CV death compared to placebo treatment. In addition, in the EMPA-REG OUTCOME and LEADER trials, the treatment group showed a reduced incidence of CV death in comparison to placebo [49, 51, 53, 54].
- *Fatal/non*-*fatal myocardial infarction* An important CV outcome to measure given the increased MI risk implied by diabetes [55], therefore its inclusion in all primary composite MACE end-points. Data from the six trials published to date has shown that all glucose-lowering treatments tested are non-inferior to placebo when it comes to MI. For a more detailed comparison of hazard rates, see Table 4.
- *Stroke* In general, the third basic element of primary composite MACE end-points. So far, considering the published results of the aforementioned six trials, none of the new glucose-lowering drugs tested increases stroke occurrence in comparison to placebo. However, in EMPA-REG OUTCOME a trend towards an increased stroke incidence was reported [49]. Conversely to EMPA-REG OUTCOME, in SUSTAIN-6, a significant reduction of stroke rates was reported for patients under semaglutide in comparison to the placebo group [52]. For more data on hazard rates, see Table 4.
- *Hospitalization for UA* The importance of this end-point varied among trials. While TECOS and ELIXA included UA in their primary end-points; and SAVOR-TIMI, EMPA-REG OUTCOME and EXAMINE included it as part of the secondary composite end-point, LEADER and SUSTAIN-6 included it as part of an extended primary composite end-point. As it happened with MI or stroke risk, UA rates did not increase under any of the treatments investigated when compared to placebo. Extended information is available on Table 4.
- *Hospitalization for HF* As shown in Table 4, rates of hospitalization for HF did not differ between placebo and treatment groups in the EXAMINE, TECOS, ELIXA or SUSTAIN-6 trials, and LEADER showed a non-significant decrease of hospitalization for HF in patients treated with liraglutide [51]. Yet, treatment with saxagliptin (SAVOR-TIMI) was found to increase hospitalization rates for HF (3.5 vs. 2.8 %; HR 1.27; 95 % CI 1.07–1.51; p = 0.007). This effect was independent of age, as confirmed by a later analysis on efficacy and safety in older patients [43]. Conversely, in EMPA-REG OUTCOME treatment with empagliflozin reduced the number of patients hospitalized for HF (2.8 vs. 4.5 %; HR 0.61; 95 % CI 0.47–0.79; p < 0.001) and improved other HF outcomes like the composite endpoint of CV death or hospitalization for HF (5.7 vs. 8.5 %; HR 0.66; 95 % CI 0.55–0.79; p < 0.001) [45, 53].
- *Serious hypoglycemic events* As part of the serious adverse event report, the rate of serious hypoglycemic events suffered by patients under treatment with the new glucose lowering drugs was investigated. Even though rates were similar to placebo in all CVOTs, and major hyperglycemia events did not differ between saxagliptin (SAVOR-TIMI) treatment and placebo, hypoglycemia occurrence generally increased with saxagliptin in combination with sulphonylureas or insulin. This effect was consistent across all age ranges analyzed [43]. On the contrary, treatment with liraglutide reduced severe hypoglycemic events in comparison to placebo (rate ratio: 0.69; 95 % CI 0.51–0.93; p = 0.02), which might be due to a reduced need for insulin co-therapy [51]. In SUSTAIN-6, the rates of severe hypoglycemia did not significantly differ between the two semaglutide-dose treatment groups and placebo [semaglutide 0.5 mg and 1.0 mg 191 (23.1 %) and 178 (21.7 %), respectively], placebo 0.5 and 1.0 mg [177 (21.5 %) and 173 (21.0 %)] [52].
- *Pancreatic effects* Regarding the possible association between incretin-based therapies and adverse pancreatic effects, CVOTs evaluated whether these new antihyperglycemic agents increased the risk for pancreatitis. Acute pancreatitis occurred slightly more often in the treatment groups than with placebo when employing saxagliptin (SAVOR-TIMI), sitagliptin (TECOS), alogliptin (EXAMINE) or lixisenatide (ELIXA), and even when no significant differences between groups could be found, a meta-analysis on trials on DPP-4 inhibitors showed a marginally higher risk of pancreatitis associated with DPP-4 treatment [56]. In LEADER and SUSTAIN-6, incidence of pancreatitis was lower, even if not statistically significant, in the intervention group than in the placebo group [51, 52].
- *Renal events and/or microvascular effects* Definitions for renal events were different for each trial. While in the ELIXA and TECOS trials there is no specification of the type of renal events [44], in EXAMINE only initiation of dialysis is reported [47]. A broader renal end-point including doubling of creatinine level, initiation of dialysis, renal transplantation or creatinine >6.0 mg/dl was used in the SAVOR-TIMI trial [43]. Regardless of end-point definition, none of these trials found differences between treatment and placebo with respect to renal function. Moreover, a further examination of the EMPA-REG OUTCOME trial regarding renal outcomes, found that addition of empagliflozin to standard treatment was associated with a slower progression of kidney disease (empagliflozin HR 0.61; CI 95 % 0.53–0.70; p < 0.001) and lower rates of clinically relevant renal events than placebo [57]. In the LEADER trial, a composite renal and retinal microvascular outcome was investigated. The renal outcome involved the new onset of macroalbuminuria or the doubling of the serum creatinine level and an eGFR ≤45 ml/min/1.73 m^2^, the need for continuous renal-replacement therapy or death from renal disease. The incidence of the composite microvascular outcome was lower with liraglutide, mainly due to a significantly lower rate of nephropathy events (HR 0.78; 95 % CI 0.67–0.92; p = 0.003) [51]. In SUSTAIN-6, the investigated renal outcome was defined as new or worsening of nephropathy and consisted on persistent macroalbuminuria, persistent doubling of the serum creatinine level and eGFR ≤45 ml/min/1.73 m^2^, or the need for continuous renal-replacement therapy. Based on that definition, semaglutide treated patients had a significantly lower risk than placebo treated patients (3.8 vs. 6.1 %, respectively; HR 0.64; 95 % CI 0.46–0.88; p = 0.005). Conversely, and somehow unexpectedly, retinopathy-derived complications (blindness, vitreous hemorrhage, or conditions requiring treatment with an intravitreal agent or photocoagulation) were significantly more often reported in the treatment group as in the placebo (3.0 vs. 1.8 %, respectively; HR 1.76; 95 % CI 1.11–2.78; p = 0.02) [52].

In general, the previous analysis shows that new glucose lowering drugs comply with FDA/EMA requirements for CV safety regardless of class. Moreover, some of them like empagliflozin, liraglutide or semaglutide even demonstrated beneficial effects over CV death risk, stroke and/or HF risk [49, 51–53].

## Discussion

CVOT trials completed after 2008 showed that new glucose lowering agents like the DPP-4 inhibitors saxagliptin, alogliptin, and sitagliptin and the GLP-1 receptor agonist lixisenatide are safe with respect to CV outcomes in high CV risk patient populations with long T2D duration (for more details on patient selection, see Table 2) under standard care for both CVD and diabetes. In addition, the LEADER study has shown that liraglutide, a GLP-1 receptor agonist, is not only safe but that is also capable of reducing CV risk and the incidence of cardiovascular-related death [51]. Furthermore, recently published results from SUSTAIN-6 have proven another GLP-1 receptor agonist, semaglutide, superior to placebo in reducing the risk of a cardiovascular composite primary end-point, driven by a significant reduction of stroke risk [52]. Moreover, treatment with the SGLT-2 inhibitor empagliflozin was not only non-inferior to placebo but also significantly reduced CV risk -as shown by the composite primary and secondary outcomes- and a composite outcome of HF hospitalization and CV death [53, 54].

Regardless of the CV safety of all anti-hyperglycemic agents tested, one trial on DPP-4 inhibitors, SAVOR-TIMI, found a significantly higher risk for HF in the treatment group and another, EXAMINE a trend towards such outcome. In contrast, there were no such concerns in the TECOS trial. Differences to baseline patient characteristics, as well as to trial design make it difficult to compare results from these trials. Moreover, the molecular structure differs among DPP-4 inhibitors and so does their safety profile. As a result, the FDA recently issued a safety warning on saxagliptin and alogliptin increasing the risk of heart failure, particularly in patients who already have heart or kidney disease [58]. Despite recent meta-analyses of randomized clinical trials including results of SAVOR-TIMI and EXAMINE suggested an increased risk of hospitalization due to HF in T2D patients [59–62], others have found no difference in hospitalization rates for HF between treatment with saxagliptin compared with sitagliptin or with DPP-4 inhibitors compared with other classes of anti-diabetes agents [63, 64].

The analyses of results of the aforementioned CVOTs have been very useful for treatment decision-making and patient safety in diabetes [65]. Not only were these trials capable of proving CV safety, but three of them, EMPA-REG OUTCOME, LEADER and SUSTAIN-6 showed cardiovascular benefits even when they were primarily designed for non-inferiority. However, it is important to note that these results are so far only valid for the particular patient groups enrolled in the studies, and that it is not clear how translatable they are to the general patient population. Furthermore, a comparison among results from CVOT is overall difficult, among other reasons because the definition of CVD risk and/or CVD is different for each trial, and with it the degree of severity of prior disease of enrolled patients highly variable. Other reasons limiting comparability among CVOTs, especially in terms of event rates, apart from the aforementioned differences in baseline patient characteristics, are the variable trial duration and the diverse definitions of the primary end-point. In addition, another obstacle for compared evaluation of trials evaluating cardiovascular outcomes before and after FDA 2008 regulation is that the routine care background from those trials is somehow dissimilar. In general, despite the great advance for the clinical practice meant by new CVOTs, there is still room for improvement [66, 67]. Trial design could still benefit from the introduction of new strategies to improve the applicability of trial results to daily clinical practice, as was agreed by the members of the first CVOT Summit of the Diabetes and CVD (D&CVD) EASD Study Group [68].

Among the recommendations stand the necessary consensus on primary end-point definition, which should be a 3-point MACE comprising cardiovascular death, non-fatal MI and non-fatal stroke. Another important point is that these cardiovascular outcomes differ greatly in their pathophysiology: while MI has a thrombotic origin [69], CV death results mostly from arrhythmia [70] and stroke can either be a product of thrombotic origin or hemorrhagic [71, 72]. These differences should be taken into account when designing and analyzing composite MACE end-points, because a positive/neutral effect in one of the components does not necessarily mean an improvement in the others, especially when considering their particular pathophysiology, as exemplified by the results of the various components of the primary composite end-point in EMPA-REG OUTCOME [49, 53]. Moreover, and especially regarding the disparate results on HF risk in DPP-4 inhibitor trials, HF risk should be investigated more closely by CVOTs [68, 73].

A major issue of CVOT design to date is patient selection criteria. Disease duration is a potential confounding factor that is not sufficiently controlled [74]. On the other hand, extrapolating CV results from this patient population to a broader one can be challenging, especially in case of superiority to placebo. To solve this matter, potential solutions could be: increasing patient retention/adherence to treatment over longer follow-up periods, promote large-scale patient enrolment by involving patient advocacy groups and modifying trial design to new approaches that minimize patient numbers and provide closer to real-world data in the standard health care system [6, 68].

Another limitation of the present CVOTs is that trial duration is too short to evaluate real-life, long-term outcomes [74], plus incurring in an unjustified high cost per patient given the limited results they provide. The extreme cost of CVOTs make them only accessible to industry and hinders an independent CV risk review [6]. To increase the amount of available data by enabling extensive follow-up and reduce trial related costs, an alternative would be to make use of comprehensive electronic health record databases with extended functionality [75, 76].

Maintaining glycemic equipoise, by addition of the test agent to standard care, has resulted in general in modest HbA1c reductions, which combined with the short follow-up time of most studies, makes it hard to positively ascertain CV benefit of these glucose-lowering drugs [40]. On the other hand, maintaining glycemic equipoise but aiming to longer follow-up times might still result in CV improvement by incidental effects from these drugs other than glycemic control, as was the case in the EMPA-REG OUTCOME trial.

Most CVOTs started after the 2008 FDA/EMA guideline analyze drugs of the SGLT-2 inhibitor, DPP-4-inhibitor, or GLP-1 receptor agonist class. Even when the ORIGIN trial already focused on the evaluation of insulin gargline versus standard care [19], since the FDA mandate only one CVOT study is investigating CV risks of insulin treatment, the ongoing DEVOTE trial on insulin glargine versus insulin degludec. To date there is not a single CVO trial on metformin or sulphonylurea alone. Considering that metformin is a first line treatment for T2D [77] and that sulphonylurea and insulin are also very common therapeutic tools in diabetes [78], more CVOTs on these drugs are essential.

Furthermore, present CVOTs are usually simple, placebo controlled, non-inferiority trials and generally lacking of head to head comparisons. Exceptionally, the ongoing CAROLINA trial includes a head to head comparison of safety issues of linagliptin, a DPP-4 inhibitor, versus a sulphonylurea (glimepiride) [79]. In the future, trial design should be aimed at matching results of several different treatment versus a reduced placebo group and ideally, under usual care. This strategy will not only allow for a direct treatment comparison but also enable a better assessment of treatment heterogeneity and possible drug interactions in the real population under standard of care [6, 68]. This strategy was followed by a recently terminated cohort study comparing head to head CV safety of GLP-1 receptor agonists to DPP-4 inhibitors, sulfonylureas, or insulin in addition to metformin, in a similar fashion to real-world conditions [80].

Despite including analysis of adverse outcomes other than CV risks, in the future a more thorough examination of microvascular complications, renal, kidney and pancreatic effects as well as cancer occurrence should be an integral component of a CVOT design [68]. Already a number of trials have been designed with this concept in mind. For instance, an ongoing clinical trial (NCT02380521) examines the effect of exenatide once weekly, a GLP-1 receptor agonist, on several CV risk markers like subclinical atherosclerosis, endothelial dysfunction, oxidative stress and atherogenic lipoproteins, which are also indicative of potential microvascular complications [81]. The ongoing CARMELINA trial (NCT01897532) also aims to characterize renal microvascular outcomes of linagliptin (DPP-4 inhibitor) on T2D patients at high CV risk. Moreover, another ongoing trial (CANVAS-R), focuses on the renal outcomes of canagliflozin (a SGLT-2 inhibitor) treatment on T2D patients at risk for CVD [82].

## Conclusion

Since the 2008 FDA/EMA regulations demanded an investigation of CV outcomes for newly developed glucose-lowering agents, a number of CVOTs have been completed and their results published. These trials, in general, have shown that glucose-lowering drugs do not increase CV risks over placebo levels, and even that some drugs, as empagliflozin, semaglutide or liraglutide, can actually lead to cardiovascular protection. However, despite satisfying the requirements of regulatory agencies when it comes to demonstrating not incrementing CV risk beyond a certain safety level, current CVOTs suffer still from certain design flaws that hinder their potential. Head to head comparisons, broader patient population groups, long-term analysis and an expansion of safety end-points, etc. would serve to improve CVOT design and expand its applicability spectrum.

## Abbreviations

CVOT: cardiovascular outcome trial
CV: cardiovascular
CVD: cardiovascular disease
T2D: type 2 diabetes
MI: myocardial infarction
CI: confidence interval
MACE: major adverse cardiovascular event
UA: unstable angina
HR: hazard ratio
EMA: European Medicine Agency
FDA: Federal Drug Agency
GLP-1: glucagon-like-peptide 1
SGLT-2: sodium–glucose cotransporter-2
DPP-4: dipeptidyl-peptidase-4
